# The Human Phenotype Ontology project: linking molecular biology and disease through phenotype data

**DOI:** 10.1093/nar/gkt1026

**Published:** 2013-11-11

**Authors:** Sebastian Köhler, Sandra C. Doelken, Christopher J. Mungall, Sebastian Bauer, Helen V. Firth, Isabelle Bailleul-Forestier, Graeme C. M. Black, Danielle L. Brown, Michael Brudno, Jennifer Campbell, David R. FitzPatrick, Janan T. Eppig, Andrew P. Jackson, Kathleen Freson, Marta Girdea, Ingo Helbig, Jane A. Hurst, Johanna Jähn, Laird G. Jackson, Anne M. Kelly, David H. Ledbetter, Sahar Mansour, Christa L. Martin, Celia Moss, Andrew Mumford, Willem H. Ouwehand, Soo-Mi Park, Erin Rooney Riggs, Richard H. Scott, Sanjay Sisodiya, Steven Van Vooren, Ronald J. Wapner, Andrew O. M. Wilkie, Caroline F. Wright, Anneke T. Vulto-van Silfhout, Nicole de Leeuw, Bert B. A. de Vries, Nicole L. Washingthon, Cynthia L. Smith, Monte Westerfield, Paul Schofield, Barbara J. Ruef, Georgios V. Gkoutos, Melissa Haendel, Damian Smedley, Suzanna E. Lewis, Peter N. Robinson

**Affiliations:** ^1^Institute for Medical Genetics and Human Genetics, Charité-Universitätsmedizin Berlin, Augustenburger Platz 1, 13353 Berlin, Germany, ^2^Berlin-Brandenburg Center for Regenerative Therapies, Charité-Universitätsmedizin Berlin, Augustenburger Platz 1, 13353 Berlin, Germany, ^3^Lawrence Berkeley National Laboratory, Mail Stop 84R0171, Berkeley, CA 94720, USA, ^4^The Wellcome Trust Sanger Institute, Wellcome Trust Genome Campus, Hinxton, Cambridgeshire CB10 1SA, UK, ^5^Department of Medical Genetics, Cambridge University Addenbrooke’s Hospital, Cambridge CB2 2QQ, UK, ^6^Université Paul Sabatier, Faculté de Chirurgie Dentaire, CHU Toulouse, France, ^7^Centre for Genomic Medicine, Central Manchester University Hospitals NHS Foundation Trust, Manchester Academic Health Sciences Centre (MAHSC), Manchester, UK, ^8^Centre for Genomic Medicine, Institute of Human Development, Faculty of Medical and Human Sciences, University of Manchester, MAHSC, Manchester M13 9WL, UK, ^9^Institute of Genetic Medicine. Newcastle University, Central Parkway, Newcastle upon Tyne, NE1 3BZ, UK, ^10^Department of Computer Science, University of Toronto, Ontario, Canada, ^11^Centre for Computational Medicine, Hospital for Sick Children, Toronto, Ontario, Canada, ^12^Department of Clinical Genetics, Leeds Teaching Hospitals NHS Trust, Leeds LS2 9NS, UK, ^13^MRC Human Genetics Unit, MRC Institute of Genetic and Molecular Medicine, University of Edinburgh, Edinburgh EH4 2XU, UK, ^14^The Jackson Laboratory, Bar Harbor, ME 04609, USA, ^15^Center for Molecular and Vascular Biology, University of Leuven, Belgium, ^16^Department of Neuropediatrics, University Medical Center Schleswig-Holstein, Kiel Campus, 24105 Kiel, Germany, ^17^NE Thames Genetics Service, Great Ormond Street Hospital, London WC1N 3JH, UK, ^18^Drexel University College of Medicine, Philadelphia, PA 19102, USA, ^19^Department of Haematology, University of Cambridge and NHS Blood and Transplant Cambridge, CB2 0PT Cambridge, UK, ^20^Autism and Developmental Medicine Institute, Geisinger Health System, Danville, PA 17822, USA, ^21^SW Thames Regional Genetics Service, St George’s Healthcare NHS Trust, Tooting, London SW17 0RE, UK, ^22^Department of Dermatology, Birmingham Children’s Hospital, Birmingham, UK, ^23^Bristol Heart Institute, University of Bristol, Bristol, UK, ^24^Department of Clinical Genetics, Great Ormond Street Hospital, London and Clinical and Molecular Genetics Unit, UCL Institute of Child Health, London, UK, ^25^Department of Clinical and Experimental Epilepsy, UCL Institute of Neurology, London, UK, ^26^Cartagenia, Leuven, Belgium, ^27^Department of Obstetrics and Gynecology, Columbia University Medical Center, New York, NY 10032, USA, ^28^Weatherall Institute of Molecular Medicine, University of Oxford, John Radcliffe Hospital, Oxford OX3 9DS, UK, ^29^Department of Human Genetics, Radboud University Medical Centre, 6500 HB Nijmegen, The Netherlands, ^30^ZFIN, University of Oregon, Eugene, OR 97403-5291, USA, ^31^Department of Physiology, Development and Neuroscience, Downing Street, Cambridge CB2 3EG, UK, ^32^Department of Computer Science, Aberystwyth University, Aberystwyth, Ceredigion, SY23 3DB, UK, ^33^Department of Medical Informatics & Clinical Epidemiology, Oregon Health & Science University, Portland, OR 97239, USA and ^34^Max Planck Institute for Molecular Genetics, Ihnestrasse 73, 14195 Berlin, Germany

## Abstract

The Human Phenotype Ontology (HPO) project, available at http://www.human-phenotype-ontology.org, provides a structured, comprehensive and well-defined set of 10,088 classes (terms) describing human phenotypic abnormalities and 13,326 subclass relations between the HPO classes. In addition we have developed logical definitions for 46% of all HPO classes using terms from ontologies for anatomy, cell types, function, embryology, pathology and other domains. This allows interoperability with several resources, especially those containing phenotype information on model organisms such as mouse and zebrafish. Here we describe the updated HPO database, which provides annotations of 7,278 human hereditary syndromes listed in OMIM, Orphanet and DECIPHER to classes of the HPO. Various meta-attributes such as frequency, references and negations are associated with each annotation. Several large-scale projects worldwide utilize the HPO for describing phenotype information in their datasets. We have therefore generated equivalence mappings to other phenotype vocabularies such as LDDB, Orphanet, MedDRA, UMLS and phenoDB, allowing integration of existing datasets and interoperability with multiple biomedical resources. We have created various ways to access the HPO database content using flat files, a MySQL database, and Web-based tools. All data and documentation on the HPO project can be found online.

## INTRODUCTION

A key challenge in genomics is to understand the phenotypic consequence of genomic variation. With the advent of next-generation sequencing technologies, the challenge is no longer to generate DNA sequence data, but to interpret them. Currently, the molecular basis of roughly 3700 Mendelian diseases has been elucidated, and a similar number of named Mendelian or suspected Mendelian diseases awaits elucidation (1).

**Figure 1.**
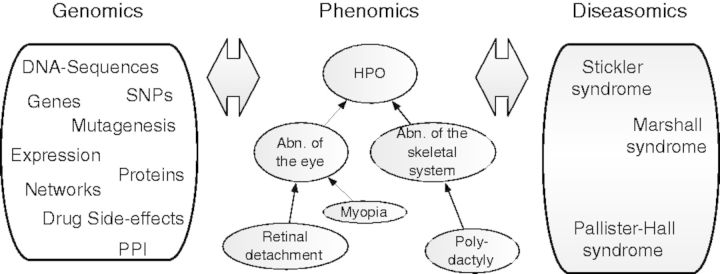
The HPO aims to act as a central resource to connect several genomics datasets with the diseasome. Thus, the HPO can act as a scaffold for enabling the interoperability between molecular biology and human disease. For example, phenotypic abnormalities in genetically modified model organisms can be mapped to human disease phenotypes (2).

The analysis of phenotypic abnormalities provides a translational bridge from genome-scale biology to a disease-centered view on human pathobiology. It is becoming clear that detailed phenotype data, combined with ever-increasing amounts of genomic data, have an enormous potential to accelerate the identification of clinically actionable complications, of disease subtypes with prognostic or therapeutic implications as well as to improve our understanding of human health and disease (Figure 1).

The description of phenotypic variation is a central topic for all of those studies, and therefore, a systematic human and computer interpretable representation is crucial due to the complex nature of human disease. Therefore, the usage of standardized measures for capturing phenotypic abnormalities is of major importance (3), but in the past, it has been difficult to agree on common semantic and technical standards as well as the ethical and legal framework for collecting and analyzing human phenotype data (4).

An ontology provides a conceptualization of a domain of knowledge, allowing communication between researchers and makes the information readable and ‘understandable’ for computers (5). The Human Phenotype Ontology (HPO) project (6) was initiated in 2007 to enable sophisticated integration of phenotype information across scientific fields and databases. Since its initial publication in 2008, the project has grown in terms of coverage, complexity, usage and cross-linking with other projects, especially from the Open Biological and Biomedical Ontologies (OBO) Foundry (7).

## THE HUMAN PHENOTYPE ONTOLOGY

The HPO covers a wide range of phenotypic abnormalities encountered in human disease (Table 1). At the time of this writing, the HPO contains 10 088 classes (terms) with 13 326 subclass relationships between those classes. The ontology is organized as three independent subontologies that cover different categories; the *mode of inheritance*, the *onset and clinical course* and the largest category of *phenotypic abnormalities*.

**Table 1.** Different types of phenotypic abnormalities covered by the HPO

‘Class’ of phenotype	HPO example
Morphological abnormality	Arachnodactyly (HP:0001166)
Abnormal process (organ)	Epistaxis (HP:0000421)
Abnormal process (cellular)	Abnormality of Krebs cycle metabolism (HP:0000816)
Abnormal laboratory finding	Glycosuria (HP:0003076)
Electrophysiological abnormality	Hypsarrhythmia (HP:0002521)
Abnormality by medical imaging	Choroid plexus cyst (HP:0002190)
Behavioral abnormality	Self-mutilation (HP:0000742)

**Figure 2.**
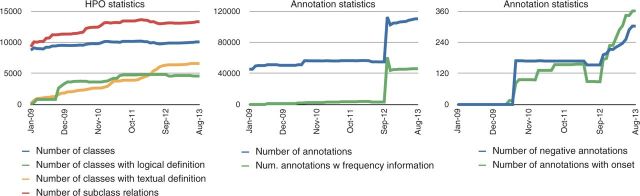
Statistics of the data from the HPO project from January 2009 to August 2013. Ontology statistics shows quantities related to the file hp.obo. The annotation statistics clearly demonstrates the inclusion of Orphanet data in October 2012.

Each class of the HPO has a unique and stable identifier (e.g. HP:0002145), a label and a list of synonyms. Most (6603, 65%) of the classes are accompanied by a detailed textual definition created by clinical experts (Figure 2).

Additionally, HPO classes now contain one or more references to other resources to promote interoperability among different biomedical research areas. As such, 39% (3956) of the HPO terms contain cross-references, with 98% of the references pointing to Unified Medical Language System and Medical Subject Headings, references that are especially helpful for linking to resources such as the Disease Ontology (8). Other cross-references include the International Classification of Diseases 10^th^ revision and the European Paediatric Cardiac Coding list. Furthermore, flat files are made available that map HPO terms to other phenotype vocabularies such as Orphanet’s *Signs and Symptoms* (see Section *HPO resources and workflow*).

To achieve semantic interoperability with other ontologies from the OBO Foundry (7), the HPO project began in 2009 to create logical definitions for each HPO class. At the time of this writing, we have created these definitions for 46% (4591) of all HPO classes. These logical axioms define the phenotypic abnormalities based on classes from other OBO Foundry ontologies (e.g. anatomy, Gene Ontology process or cell type). They are formal descriptions, that are machine processable and usable for automated logical inference and reasoning (9,10). For example, we have created the following logical definition of the HPO term *Hypoglycemia* (shown in Manchester syntax):

Class: Hypoglycemia
EquivalentTo:
 ‘decreased concentration’
and towards some ‘glucose’
and inheres_in some ‘portion of blood’
and qualifier some ‘abnormal’

Here, term identifiers are skipped and only term labels are shown for the purposes of readability. In this example, the class *Hypoglycemia* is defined as being equivalent to the intersection of all classes of things that are ‘A concentration which is lower relative to the normal’ (*decreased concentration* from PATO); ‘deviate from the normal or average’ (*abnormal* from PATO), with respect to (towards) glucose and inhering in ‘blood’ [using the term *portion of blood* from the Foundational Model of Anatomy (11)]. Defining ontology terms in this way assists in automating ontology construction, and provides a tool for integrative computational analysis of human and model organism phenotypes against the background of the knowledge incorporated in ontologies such as Gene Ontology, Foundational Model of Anatomy and Chemical entities of biological interest (ChEBI) (12–15).

## PHENOTYPE ANNOTATION DATA

We provide a large set of phenotype annotations, i.e. statements that link a particular term from the HPO to specific diseases or genes. These annotations are made for the most specific term of the HPO, as all of the ancestor terms are implicitly annotated as well.

At the time of this writing, we provide 110 301 annotations to 7354 diseases listed in the Online Mendelian Inheritance in Man [OMIM, (16)] database, Orphanet (17) and DECIPHER (18). On average, each disease entry has 15 HPO annotations. For Orphanet entries that are exactly mapped to one OMIM entry, we merge the entries and record the provenance of the annotations.

The annotations of OMIM entries are a mixture of manual annotations performed by the HPO team and automated matching of the OMIM *Clinical Synopsis* to HPO term labels. The substantial increase in annotation data during since 2008 is shown in Figure 2.

Each annotation may have several meta-attributes such as the age of onset, the frequency or a modifier. At the moment, 46 149 annotations have information on the frequency with which individuals with a given disease have a certain phenotypic feature. For instance, 9 of 43 persons with the disease *sialidosis type II* have *cherry red spot of the macula* (HP:0010729) (19). At the moment, the majority of frequency annotations are derived from Orphanet, but a growing number is based on the manual annotation efforts by the HPO team. Furthermore, we provide a set of 303 negative annotations (NOT-modifier), for which patients with this disease are known not to have the clinical feature in question. The frequency and negation information may be important for the differential diagnosis (20). For 361 annotations, details on the onset are provided. Note that the onset-information may apply to a disease (e.g. Marfan syndrome has *congenital onset*) or to a single phenotype annotation (e.g. *Kyphosis* in Hurler syndrome (OMIM:607014) has the meta-annotation *childhood onset*).

Ontologies such as the HPO are not designed to capture quantitative information such as a blood glucose level of 146 mg/dl or an adult body height of 147 cm. Instead, HPO terms often express qualitative information about an excess or a reduction in quantity of the entity in question (i.e., *Hypoglycemia* and *Tall stature*). For some clinical manifestations, however, it has been found to be clinically useful to divide an entity into two or more categories. For instance, the degree of intellectual disability is often reported as one of the four categories Mild, Moderate, Severe and Profound. In these cases, the HPO aims to follow common clinical usage and provide corresponding terms defined according to clinical norms. Additionally, modifiers such as *episodic* or *recurrent* are possible. A summary of meta-annotations and their definitions can be found in Table 2.

**Table 2.** Meta-information for HPO phenotype annotations

Meta-attribute	Possible values (explanation in brackets)
Qualifier/Modifier	not, mild (±2–3 SD from mean), moderate (±3–4 SD from mean), severe (±4–5 SD from mean), profound (±5SD and greater from mean), secondary, chronic, (non)progressive, episodic, recurrent, bilateral, unilateral, distal, proximal, refractory and generalized
Evidence Code	ITM (inferred by text mining), IEA (inferred from electronical annotation), PCS (published clinical study), ICE (individual clinical experience), TAS (traceable author statement)
Onset modifier	Any term from HPO-subontology *Age of onset*
Frequency modifier	percentage value (e.g. 25%), n of m (e.g. 3/10 patients), very rare, rare, occasional, frequent, typical, variable, common, hallmark and obligate

The meaning/definition of the values is shown in brackets. (SD = standard deviation).

## CLINICAL INTEGRATION AND USE

The HPO project is collaborating with many clinical groups to refine and extend current terms and annotations. A major effort was undertaken in 2012 with clinicians from the Deciphering Developmental Disorders (21) project to ensure that HPO reflects the needs of that project. Efforts were made to eliminate redundancies and to fill in gaps in the HPO coverage of organ systems, metabolism, neoplasms, neurology and behavior. Among other things, the Onset section of the HPO was revised to provide a small set of well-defined and non-overlapping terms based on published recommendations (22) (Table 3). Input and collaboration from other clinical groups will be welcomed.

**Table 3.** Definitions of age-of-onset terms in the HPO

Onset of manifestations	Definition
Less than 1 year	
Embryonal	<8 weeks’ gestation
Fetal	8 weeks’ gestation–birth
Neonatal	Birth–28 days
Infantile	28 days–1 year
More than 1 year	
Childhood	1–5 years
Juvenile	5–15 years
Adults	
Young adult	<40 years
Mid adult	40–60 years
Old age	>60 years

Whole-exome sequencing (WES) is accelerating the pace of discovery of novel Mendelian disease genes, but many challenges remain. A standard strategy for WES data analysis is to compare variants found in multiple affected patients. Especially with autosomal dominant disorders, many unrelated individuals must be analyzed for this strategy to be successful (23). Therefore, one of the first tasks in WES disease gene discovery projects is to identify multiple patients with the same disease phenotype, which has been extremely successful in identifying novel disease genes even in diseases for which there was little or no previous knowledge about the characteristics of the disease gene. However, many of the Mendelian diseases still waiting to be discovered are very rare or difficult to diagnose clinically. To make progress on elucidating these disorders, it will likely be necessary to combine data from multiple centers to identify a sufficient number of patients with mutations in the same gene and comparable phenotypes—which is widely accepted as a necessary criterion for the identification of a novel disease gene.

This approach has been implemented successfully for copy-number variation (CNV) disorders in the International Standards for Cytogenomic Arrays Consortium’s publicly available database of CNVs identified during the course of routine clinical microarray testing (http://www.ncbi.nlm.nih.gov/dbvar/studies/nstd37/;https://www.iscaconsortium.org). Recognizing that cataloging the phenotype information associated with each CNV would be key in trying to elucidate genotype–phenotype relationships, the group began using HPO terms (as opposed to free text) to describe the phenotypes in a manner that was generalizable (to maintain patient anonymity) as well as easily indexable and searchable for the clinical and research communities (24). Given the success of this approach, the International Standards for Cytogenomic Arrays has expanded its focus to include sequence variation, and, under the name International Collaboration for Clinical Genomics, will continue to use HPO terms to describe the phenotypes associated with results from additional testing modalities, including WES (25).

A similar approach is also being used by the DECIPHER project, which enables clinical scientists worldwide to maintain records of phenotype and chromosome rearrangement for their patients and, with informed consent, share this information with the wider clinical research community to find clusters of rare cases having phenotype and structural rearrangement in common (18). The Deciphering Developmental Disorders project of the Wellcome Trust Sanger Institute has been initiated to use new genomic technologies including especially WES to identify novel etiologies for developmental disorders, and is focused on severe and extreme developmental phenotypes affecting any organ system, which are coded using HPO.

An international collaborative study, the Biomedical Research Centres/Units Inherited Diseases Genetic Evaluation consortium, will use the HPO database to record detailed clinical phenotypes of patients with rare inherited disorders (www.bridgestudy.org). The HPO database that comprises phenotypes related to abnormalities in blood and blood-forming tissues has already facilitated detailed description of the clinical phenotypes of patients with bleeding and platelet disorders (Biomedical Research Centres/Units Inherited Diseases Genetic Evaluation-Bleeding and Platelet Disorders). The homogenization of these clinical phenotypes related to bleeding and platelet disorders will further assist in the clustering of data for detailed bioinformatics analysis of exome sequence data. These patients will be part of the NIHR Bioresource for Rare Diseases.

The European Cytogeneticists Association Register of Unbalanced Chromosome Aberrations (ECARUCA, http://www.ecaruca.net), initiated in 2003, is an online database that collects and provides detailed, curated clinical and molecular information on rare unbalanced chromosome aberrations that are considered to be likely causative for the patient’s phenotype (26). The objective of ECARUCA is to improve the knowledge of rare chromosome aberrations both for medical and research purposes. Currently, the database contains more than 4800 cases with HPO features characterizing these cases, and all these data are publicly available to professionals in genetics.

The Nijmegen Genetics Phenotype Database (NGPD, https://www.clinicalfeatures.eu/default.aspx) aims to collect detailed phenotype information of patients with unexplained intellectual disability and/or congenital anomalies using the HPO. The goal of the NGPD is to identify patients who have similar clinical features that are likely due to the same or a related genetic defect. The NGPD currently contains more than 8000 patients with 73 496 HPO features annotated to these patients (median seven features per patient). Computational approaches are currently being developed for the identification of clusters of phenotypically overlapping patients. Exome sequencing and targeted candidate gene analysis will ultimately provide a diagnosis for many of these patients.

Cartagenia (www.cartagenia.com), a genetics software solution provider that services diagnostic laboratories through a set of automated tools for variant interpretation, filtration, reporting and sharing, has standardized the phenotype functions for clinical patient record annotation of its BENCH laboratory platform on HPO. Several advantages come by using HPO: automated genotype–phenotype correlation, advanced search of patients within laboratories but also in external databases (see earlier) and easy sharing of patient phenotype data among different consortia.

Interoperability between laboratories sharing case information has benefited from standardization on HPO. With more than 120 laboratories and clinics using Cartagenia BENCH in a routine setting, a number of consortia have emerged where not just genotype but also phenotype data are shared. Examples include a number of national consortia sharing variants and phenotype data (The Netherlands, France, UK and Norway) as well as disease-specific registries for (autism, primary immune deficiencies and cardiogenetics), ECARUCA, large prenatal case registries such as the UK-led NHS EACH study and a US-led study at the Columbia University, which have set the phenotyping standard for other prenatal genotype–phenotype registries.

## HPO WORKFLOW AND RESOURCES

As mentioned before, we use a continuous integration system (Hudson) for the management of stable releases of the HPO-related data (27) to ensure that users are provided with up-to-date and validated resources. To achieve this, only stable builds are made public, and any curation errors that lead to build failures are detected by our software and prevented from being propagated onto the public Web site. For different aspects of the data, we have generated different jobs and an overview of the job organization can be found in Table 4. The major focus is the phenotype ontology and the annotation data, but closely related projects such as the cross-species phenotype ontology Uberpheno (13) are available as well.

**Table 4.** Content of and access to the stable releases of the data provided by the HPO project

Release category	URL of latest stable release for job (relative to http://compbio.charite.de/hudson/job/)	File(s) at URL	File description
HPO releases	hpo/lastStableBuild/	hp.obo, hp.owl	HPO in OBO/OWL format as generated by Oort.
		human-phenotype-ontology_xp.obo	Logical definitions of HPO terms.
		onet_hpo.tsv, LDDB2HPO-v2.csv, medraMapping.tsv	Mappings to other phenotype vocabularies, e.g. Orphanet, LDDB, MedDRA.
Disease annotations	hpo.annotations/lastStableBuild/	negative_phenotype_annotation.tab	Disease-HPO term associations asserted not to be associated with the corresponding disease.
		phenotype_annotation_hpoteam.tab	Manual and semi-automatic annotations of syndromes from OMIM and DECIPHER.
		phenotype_annotation.tab	Manual and semi-automatic annotations of OMIM and DECIPHER augmented with annotations to Orphanet syndromes.
Other data	hpo.annotations.monthly/lastStableBuild/	<*source* >_ <*freq* >_genes_to_phenotype.txt	Mapping of human genes to phenotypic features (via disease-to-gene relationship). (<*source* > is one of ALL_SOURCES, OMIM, or ORPHANET; <*freq* > is either ALL_FREQ or TYPICAL).
		<*source* >_ <*freq* >_phenotype_to_genes.txt	Mapping of phenotypic features to human genes (via disease-to-gene relationship). (<*source* > is one of ALL_SOURCES, OMIM, or ORPHANET; <*freq* > is either ALL_FREQ or TYPICAL).
		MYHPO_MM_YYYY.sql	MySQL dump of the HPO database, where MM and YYYY denote the month and year of release.
	hpo.diseasesimilarity/lastStableBuild/	matrices.tar.gz	Precomputed disease–disease similarity matrix for all diseases with annotations to HPO’s phenotypic abnormality subontology. Symmetric and asymmetric semantic similarity score.
	hpo.ontology.uberpheno/lastStableBuild/	crossSpeciesPheno.obo	Cross-species phenotype ontology (human, mouse, zebrafish).
		HSgenes_crossSpecies PhenoAnnotation.txt	Annotation of all human genes to terms in crossSpeciesPheno.obo (uses orthology to human genes obtained from MGI and ZFIN). See (13).

The HPO release (job hpo) is triggered whenever changes in any of the ontology or logical definition files are uploaded. For every build, the OBO Ontology Release Tool (Oort, https://code.google.com/p/owltools/wiki/OortIntro) is used to generate OBO- and OWL-format versions of the HPO. In addition, the GULO software (28) is used to generate a report on the overlap between the hierarchy inferred from the logical definitions and the manually asserted HPO hierarchy. This is used to incrementally improve both the logical definitions and the HPO structure.

Annotation data are also integrated in our Hudson build system (Table 4). Every HPO release induces a rebuild of the annotation data (job hpo.annotations). This job pulls the latest manual annotation data (http://svn.code.sf.net/p/obo/svn/phenotype-commons/annotations/OMIM/by-disease/annotated/) and the latest Orphanet data (http://www.orphadata.org) and constructs one integrated disease annotation file. Again only successful builds are made available, such that e.g. manually curated annotations are automatically checked for consistency before being offered to the public. The simplest check verifies the syntactical correctness of the input files. Another example is that the generation of annotation files fails if there are annotations to obsolete terms, which are terms that have been marked as to be replaced by other HPO terms and thus should not be used for annotation anymore. Another check confirms that annotation onset-modifiers are correctly chosen from the *Onset and clinical course* subontology.

Once a month, several secondary files are created automatically by the Hudson build system. The job hpo.annotations.monthly creates an MySQL version of the HPO and the annotation data. It also constructs direct gene-to-phenotype mappings, which use known gene-to-disease relations (from morbidmap and Orphanet) and disease-to-phenotype relations from the job hpo.annotations. So for example the gene *ATXN10* (Entrez ID 25814) will be associated with *Gait ataxia* (HP:0002066), because mutations in that gene cause *Spinocerebellar ataxia* (OMIM:603516), which is annotated to this HPO class. The files are constructed for different phenotype annotation sources (OMIM, Orphanet) and different frequency thresholds.

Other jobs generate the data used by the Phenomizer (hpo.annotations.monthly.phenomizer), a precomputed disease–disease similarity matrix (hpo.diseasesimilarity), as well as the cross-species phenotype resource Uberpheno (hpo.ontology.uberpheno).

Besides these files, the information of the HPO project can also be accessed in other ways. The HPO Web site offers an individual page for each HPO term (e.g. http://www.human-phenotype-ontology.org/hpoweb/showterm?id=HP:0000127), each of which displays the term label, synonyms, definition and links to genes and diseases. The PhenExplorer is a Web-based application that offers much of the same functionality in a graphical user interface. The HPO is being increasingly used as a basis for integrating phenotypic abnormalities into computational algorithms for diagnostics and research. For instance, Phenomizer (29) and BOQA (20) can be used to assist clinical differential diagnostic for human genetics, and MouseFinder (30), Monarch (http://monarchinitiative.org) PhenoDigm (14) as well as PhenomeNET (12) enable searches for novel disease genes based on the analysis of model-organism phenotypes. The HPO has been used to integrate phenotypic information into computational analysis of the distribution of proteins in the postsynaptic density of the human neocortex (31), to derive a disease–disease similarity measure for the prediction of novel drug indications (32) and to analyze overrepresentation of phenotypes associated with individual protein domains (33). A summary of tools and applications using data from the HPO project is given in Table 5.

**Table 5.** Tools and applications using HPO

Tool	Reference/URL
*Differential diagnosis and exome analysis*	
Phenomizer	(29)
BOQA	(20)
Exomiser	http://www.sanger.ac.uk/ resources/databases/exomiser/
*Clinical data management and analysis*	
Cartagenia	http://www.cartagenia.com/
ECARUCA	(26)
DECIPHER	(18)
PhenoTips	(34)
*Cross-species phenotype analysis*	
PhenoDigm	(14)
MouseFinder	(30)
Monarch	http://monarchinitiative.org
PhenomeNet	(12)
Uberpheno	(13)

The HPO project offers a number of files that are intended to help users use these kinds of data for their own research. A Hudson job (hpo.diseasesimilarity) creates a precomputed disease similarity matrix, which contains all diseases that have annotations to the HPO subontology ‘phenotypic abnormality’. The similarity value between two diseases is calculated using the HPO annotations for the diseases to calculate a semantic similarity measure (6). A symmetric and an asymmetric version of the disease similarity matrix are calculated (29,35).

The HPO tracker at http://purl.obolibrary.org/obo/hp/tracker can be used to request new classes or to suggest structural changes of the HPO subsumption hierarchy.

Classes of the HPO and associated diseases and genes can be accessed using persistent URLs of the form http://purl.obolibrary.org/obo/HP_ID, where <ID> represents the numeric identifier of the HPO class. Further information on HPO-related publications and general announcements can be found on the HPO Web site at http://www.human-phenotype-ontology.org.

## FUTURE DEVELOPMENTS

Development of the HPO has continued apace since its initial publication in 2008 (6). The HPO has focused on providing a well-defined, comprehensive and interoperable resource for computational analysis of human disease phenotypes and has been used as a basis for a wide panoply of tools to perform analysis in clinical and in research settings. While the initial focus of the HPO was placed on rare, mainly Mendelian diseases, HPO annotations are now available also for CNV diseases, and a pilot project to explore the development of annotations for common diseases is currently underway.

Deep phenotyping has been defined as the precise and comprehensive analysis of phenotypic abnormalities in which the individual components of the phenotype are observed and described (36). Deep phenotype analysis is an essential component of the emerging field of precision medicine, which aims to provide the best available care for each patient based on stratification into disease subclasses with a common biological basis of disease. The HPO aims to provide a powerful and manually curated resource to support efforts to discover disease subclasses, and to translate this knowledge into clinical care, by providing the means to capture, store and exchange phenotypic data. The clinical data that have been captured in this fashion are computable and can be easily integrated into computational algorithms for translational biomedical research.

## FUNDING

The Deutsche Forschungsgemeinschaft [DFG RO 2005/4-2]; Bundesministerium für Bildung und Forschung [BMBF project number 0313911]; the European Community’s Seventh Framework Programme [Grant Agreement 602300; SYBIL]. Additional support was received from the Director, Office of Science, Office of Basic Energy Sciences, of the U.S. Department of Energy under [Contract No. DE-AC02-05CH11231]; the MGD grant from the National Institutes of Health [HG000330]; the ZFIN grant from the National Institutes of Health [U41-HG002659]; National Institutes of Health [R01-HG004838 and R24-OD011883]; National Institute for Health Research University College London Hospitals Biomedical Research Centre. Funding for open access charge: Institutional support.

*Conflict of interest statement*. None declared.
